# Supplementary material for: The Role of Mycoplasma bovirhinis in the Development of Singular and Concomitant Respiratory Infections in Dairy Calves from Southern Brazil
Source: Pathogens. 2024 Jan 26;13(2):114. doi: 10.3390/pathogens13020114 (PMC10892079; doi:10.3390/pathogens13020114)
Supplement: Supplementary file 1 [file pathogens-13-00114-s001.zip › pathogens-2826321-supplementary.pdf]

The participation of *Mycoplasma bovirhinis* in the development of singular and concomitant respiratory infections in dairy calves from Southern Brazil

Ana Paula Souza Frucch [1], Alais Maria Dall Agnol [1], Eloiza Teles Caldart [2], Dalton Everton Bronkhorst [1], Alice Fernandes Alfieri [1,3,4] Amauri Alcindo Alfieri [1,3,4] and Selwyn Arlington Headley [3,4,5]*

[1] Laboratory of Animal Virology, Department of Preventive Veterinary Medicine, Universidade Estadual de Londrina, Paraná, Brazil.
[2] Laboratory of Protozoology and Parasitic Diseases, Department of Preventive Veterinary Medicine Universidade Estadual de Londrina, Paraná, Brazil.
[3] Multi-User Animal Health Laboratory (LAMSA), Department of Preventive Veterinary Medicine Universidade Estadual de Londrina, Paraná, Brazil.
[4] National Institute of Science and Technology for Dairy Production Chain (INCT–LEITE), Universidade Estadual de Londrina, Londrina, Brazil
[5] Laboratory of Animal Pathology, Department of Preventive Veterinary Medicine, Universidade Estadual de Londrina, Paraná, Brazil.

* Correspondence: e-mail selwyn.headley@uel.br; Tel.: + 55 43 3371-4766

Citation: To be added by editorial staff during production.

Academic Editor: Firstname Lastname

Received: date
Revised: date
Accepted: date
Published: date

Copyright: © 2023 by the authors. Submitted for possible open access publication under the terms and conditions of the Creative Commons Attribution (CC BY) license (https://creativecommons.org/licenses/by/4.0/).

Supplementary Table 1. Targets genes, primers, and amplicon size of the molecular assays used to identify infectious disease pathogens of respiratory and enteric diseases of cattle.

Pathogens	Target genes	Primer sequencies (5´ - 3´)	Amplicon size (bp)	Reference order
**Viral**				
OvGHV2	Tegument protein	Fw-AGTCTGGGTATATGAATCCAGATGGCTCTC<br>Rv-AAGATAAGCACCAGTTATGCATCTGATAAA	422	1
BoGHV6	DNA polymerase	Fw-ACAGACGGGCAGCAGATAAG<br>Rv-ATGGTTCGCCCCTGTAGAGT	551	2
BCoV	N gene	Fw-TTGCTAGTCTTGTTCTGGC<br>Rv-TGTGGGTGCGAGTTCTGC	251	3
BVDV	5'UTR	Fw-ATGCCC(T/A)TAGTAGGACTAGCA<br>Rv-TCAACTCCATGTGCCATGTAC	288	4
BRSV	G gene	Fw-CATCAATCCAAAGCACCACACTGTC<br>Rv-GCTAGTTCTGTGGTGGATTGTTGTC	371	5
BoAHV1	C gene	Fw-CAACCGAGACGGAAAGCTCC<br>Rv-AGTGCACGTACAGCGGCTCG	354	6
BPIV-3	HN gene	Fw-GAATGACTCATGATAGAGGTAT<br>Rv-AGGACAACCAGTTGTATTACAT<br>Rv-ATGTTGTTRATGATGGTGTTGA	647	7
**Bacterial**				
Mannheimia haemolytica	lktA-artJ intergenic region	Fw-GTCCCTGTGTTTTCATTATAAG<br>Rv-CACTCGATAATTATTCTAAATTAG	385	8
Histophilus somni	16S	Fw-GAAGGCGATTAGTTTAAGAG<br>Rv-TTCGGGCACCAAGTRTTCA	408	9
Pasteurella multocida	ORF KMT1	Fw-GCTGTAAACGAACTCGCCAC<br>Rv-ATCCGCTATTTACCCAGTGG	460	10
Mycoplasma bovis	16S-23S rRNA internal transcribed spacer	Fw-GTACACTTGTCTTTTATCACTATA<br>Rv-AAGGTATCTCGCTTTATGTCCT	488	11

References

1 Baxter, S. I., Pow, I., Bridgen, A. & Reid, H. W. PCR detection of the sheep-associated agent of malignant catarrhal fever. *Arch Virol* **132**, 145-159 (1993). https://doi.org/10.1007/bf01309849

2 Oliveira, C. H. *et al.* Bovine herpesvirus 6 in buffaloes (*Bubalus bulalis*) from the Amazon region, Brazil. *Trop Anim Health Prod* **47**, 465-468 (2015). https://doi.org/10.1007/s11250-014-0733-z

3 Takiuchi, E., Stipp, D. T., Alfieri, A. F. & Alfieri, A. A. Improved detection of bovine coronavirus N gene in faeces of calves infected naturally by a semi-nested PCR assay and an internal control. *J Virol Methods* **131**, 148-154 (2006). https://doi.org/10.1016/j.jviromet.2005.08.005

4 Vilcek, S. *et al.* Pestiviruses isolated from pigs, cattle and sheep can be allocated into at least three genogroups using polymerase chain reaction and restriction endonuclease analysis. *Arch Virol* **136**, 309-323 (1994). https://doi.org/10.1007/bf01321060

5 Vilcek, S., Elvander, M., Ballagi-Pordány, A. & Belák, S. Development of nested PCR assays for detection of bovine respiratory syncytial virus in clinical samples. *J Clin Microbiol* **32**, 2225-2231 (1994). https://doi.org/https://doi.org/10.1128/jcm.32.9.2225-2231.1994

6 Claus, M. P. *et al.* Rapid detection and differentiation of bovine herpesvirus 1 and 5 glycoprotein C gene in clinical specimens by multiplex-PCR. *J Virol Methods* **128**, 183-188 (2005). https://doi.org/10.1016/j.jviromet.2005.05.001

7 Zhu, Y. M. *et al.* Isolation and genetic characterization of bovine parainfluenza virus type 3 from cattle in China. *Vet Microbiol* **149**, 446-451 (2011). https://doi.org/10.1016/j.vetmic.2010.11.011

8 Angen, O. *et al.* Respiratory disease in calves: microbiological investigations on trans-tracheally aspirated bronchoalveolar fluid and acute phase protein response. *Vet Microbiol* **137**, 165-171 (2009). https://doi.org/10.1016/j.vetmic.2008.12.024

9 Angen, O., Ahrens, P. & Tegtmeier, C. Development of a PCR test for identification of *Haemophilus somnus* in pure and mixed cultures. *Vet Microbiol* **63**, 39-48 (1998). https://doi.org/https://doi.org/10.1016/s0378-1135(98)00222-3

10 Townsend, K. M., Frost, A. J., Lee, C. W., Papadimitriou, J. M. & Dawkins, H. J. Development of PCR assays for species- and type-specific identification of *Pasteurella multocida* isolates.

Journal of clinical microbiology **36**, 1096-1100 (1998). https://doi.org/10.1128/jcm.36.4.1096-1100.1998

11 Voltarelli, D. C. *et al.* A nested-PCR strategy for molecular diagnosis of mollicutes in uncultured biological samples from cows with vulvovaginitis. *Anim Reprod Sci* **188**, 137-143 (2018). https://doi.org/https://doi.org/10.1016/j.anireprosci.2017.11.018
